# Stress and health Huangshan-style

**DOI:** 10.1007/s12192-016-0674-8

**Published:** 2016-02-04

**Authors:** Lingjia Qian, Robert M. Tanguay, Tangchun Wu, Lawrence E. Hightower

**Affiliations:** 1 Department of Stress Medicine, Institute of Basic Medical Sciences, No. 27 Taiping Road, Haidian District, Beijing, 100850 People’s Republic of China; 2 LGCD, IBIS, Dept Molecular Biology, Medical Biochemistry and Pathology, Québec, Canada G1V 0A6; 3 School of Public Health, Huazhong University of Science and Technology, 13 Hangkong Road, Wuhan, 4300030 People’s Republic of China; 4 Molecular and Cell Biology Department, University of Connecticut, Storrs, CT 06269-3125 USA

**Keywords:** Stress responses, Biology, Medicine, Molecular chaperones

## Abstract

The Seventh International Congress of the Cell Stress Society International (CSSI) was held as a joint meeting with the newly organized committee of Stress Physiology, the Chinese Association for Physiological Sciences (CAPS). There were over 200 colleagues and their students in attendance from 22 different countries. The topics of the congress were core scientific areas in the field of stress and health. The keynote speakers were Fu-Chu He (China), E.R. (Ron) de Kloet (The Netherlands), and Kazuhiro Nagata (Japan). The CSSI Medallion for Career Achievement in the cell stress and chaperones field was awarded to Kazutoshi Mori (Japan). Twelve student post awards were given in recognition of a very high quality poster session. In the tradition of this series of congresses, cultural events were an important part of the program. In addition, participants became better acquainted during trips to the ancient shopping street, an evening at the Chinese opera, and a lesson in Tai Chi from a master. The first groups of CSSI Fellows and Senior Fellows were presented their rosettes and certificates during the congress.

**Electronic supplementary material:**

The online version of this article (doi:10.1007/s12192-016-0674-8) contains supplementary material, which is available to authorized users.

## Introduction

The Seventh International Congress was held in Huangshan City, China, from the 15th through the 19th of September 2015. The principal organizer of the congress was Professor Lingjia Qian (Beijing). Professor Qian’s laboratory has been a leading group in China for the study of the role of stress in major human diseases (Liu et al. 2014; Gao et al. 2015). The co-organizers were Professors Lawrence E. Hightower (Storrs, CT) (Verma et al. 2015; Fuller et al. 2013), Robert M. Tanguay (Quebec City) (Morrow et al. 2015a, b), and Tangchun Wu (Wuhan) (Cui et al. 2015; Chen et al. 2015). The Cell Stress Society International (CSSI) and the committee of Stress Physiology of the Chinese Association of Physiological Sciences (CAPS) sponsored the congress. Professor Qian is currently the chairman of the committee of Stress Physiology in CAPS and she was instrumental in the founding of the committee of Stress Physiology in CAPS. Leaders of the CSSI and the committee of Stress Physiology in CAPS worked together very efficiently to develop this congress.

There were more than 200 participants from at least 22 different countries, so this was a truly international meeting (see group photo of participants, Supplementary Figure 1). The topics of the congress were core scientific areas in the field of stress and health. They included physiological and psychological responses in stress biology; stress and brain networks; gene transcription under stress; activation of stress responses to prevent and treat major human diseases; the study of stress granules common to stressed cells of many types; the links between stress and exercise; the structure, regulation, and functions of small heat shock proteins many of which are still unknown; stress and women’s health; molecular chaperones and mitochondrial homeostasis; stress and immune regulation; and the links between stress and a broad range of diseases.

## Keynote speakers session

The congress opened on a high note with the first session devoted to three keynote addresses (speaker photos in Supplementary Figure 2, 3, 4). Fu-Chu He (China) gave a talk entitled “Human Liver Proteome Project, a Demo for Human Proteome Project” (Xu et al. 2015; Tong et al. 2015). E. R. (Ron) de Kloet (Holland) presented “Tipping the Balance to Stress Resilience” (de Kloet 2014; de Kloet et al. 2005). Kazuhiro Nagata (Japan) gave a talk entitled “ERdj5 and ERdj8: Regulation of Proteostasis and Calcium Homeostasis in the ER and Negative Regulation of Macro-autophagy” (Kawasaki et al. 2015; Kakugawa et al. 2013).

Dr. Fu-Chu He is a pioneer in systems approaches to protein science in China such as proteomics. He has also been a leader in international cooperation and database sharing. Because of his central role in this rapidly developing field, Dr. He has multiple professional affiliations including The State Key Laboratory of Proteomics in the Beijing Proteome Research Center at the Beijing Institute of Radiation Medicine, National Engineering Research Center for Protein Drugs, and National Center for Protein Sciences (Phoenix Center), all in Beijing, China.

Dr. He is a member of the Chinese Academy of Sciences. He has published more that 250 papers in international peer-reviewed journals and is considered the leading scientist studying proteomics in China. Dr. He is currently the president of Asia Oceania HUPO (AOHUPO), and he also is the Inaugural President of the Institutes of Biomedical Sciences, Fudan University. In his position as the chief scientist of the China Human Proteome Project, he is propelling the campaign of the National Center for Protein Sciences (Beijing)-PHOENIX in China, which opened this past October. His major fields of research are genomics, proteomics, bioinformatics, and systems biology.

Dr. He began his talk with an overview of the impressive progress that has been made over the past 18 years on the Human Liver Proteome Project (HLPP). This project was the pathbreaking initiative of the Human Proteome Organization (HUPO) to concentrate on human organs. HUPO is a global international organization with the grand scientific objective to reveal the “solar system” of the human liver proteome in all its manifestations. Dr. He compared this initiative to a rewriting of the ancient Prometheus Myth into a modern form. In Greek mythology, Prometheus was given the task to create man. The myth says that he shaped man out of mud and Athena breathed life into the clay figurine. Proteomics is reshaping our understanding of what it is to be human.

The HLPP is now the largest proteomic dataset of a human organ, allowing the establishment of the first version of the human liver proteome (HLP). Targeted studies by a variety of groups using this database have shown for example that the stress proteins HSP90 and HSP70 along with hCE1 may be therapeutic as well as being pharmacological targets or biomarkers for HBV-related diseases. A very large amount of primary biological information has been accumulated in this way. It is now accessible to all researchers via three web-based databases: Liverbase, dbLEP, and LiverMap.

The HLPP through its successes has become a model integrative approach for how to create a protein atlas for a particular organ of the body. Standard operating procedures have been developed that are being applied to other HUPO initiatives. The theory of “solar system” has gone global. In China, there is a new initiative now renamed the Chinese Human Proteome Project (CNHPP), which has been expanded to include proteins in ten major organs and body fluids.

The second speaker, Dr. E.R. (Ron) de Kloet, is an Academy Professor of the Royal Netherlands Academy of Arts and Sciences and an Emeritus Professor of Medical Pharmacology located in the Department of Endocrinology and Metabolic Diseases at the Leiden University Medical Center. He noted in his biographical sketch that “For half a century I am fascinated by the question how stress hormones, that are critical for life, can change their action in the brain from protective to harmful, while enhancing vulnerability to stress-related disorders.” This interest has resulted in more than 600 scientific articles. His research has received international recognition by the Geoffrey Harris Award of the European Federation of Endocrine Societies and the European College of Neuropsychopharmacology Award. Dr. de Kloet also received the Lifetime Achievement Award of the International Society of Psychoneuroendocrinology and the Golden Emil Kraepelin Medal for the impact of basic research on the diagnosis and treatment of depression.

Professor Ron de Kloet began his presentation by setting the stage for the multidisciplinary audience to understand the role of the adrenal hormones cortisol and corticosterone (CORT) in carrying out the signals from the hypothalamus-pituitary-adrenal (HPA) axis to exert dramatic effects on the brain circuits underlying stress adaptation. Dr. de Kloet went on to describe his group’s observations in chronic stress models of human psychopathology. They discovered a large genomic reorganization in hippocampal neurons that was revealed after an acute challenge with CORT. It was characterized by activation of the intracellular signaling pathways known to participate in chromatin organization and epigenetic processes. These stressful conditions cause a shift in resources in the brain that facilitate simple habit behavior to improve coping with stress. They further found that this switch in resources is prevented by manipulation of mineralocorticoid receptors. These studies bolster the evidence that this CORT receptor is a major target for increasing stress resilience.

The third keynote speaker, Professor Kazuhiro Nagata, is a pioneering scientist in the fields of stress responses and molecular chaperones in the endoplasmic reticulum (ER). In the early years of our field, Dr. Nagata found that one of the so-called heat shock proteins, Hsp47, is an ER-resident chaperone dedicated to the biosynthesis of collagen, i.e., it is a specific collagen-binding chaperone. He made this observation at a time when very little was known about the function of chaperones or for that matter their physiological role in organismal responses to stress of various kinds including heat shock. Professor Nagata is a founding member of the Cell Stress Society International as well as a founding member of the editorial review board of *Cell Stress & Chaperones* journal. He was co-organizer of the Fourth CSSI International Congress in Sapporo, Japan, in 2009. He was among the small group of colleagues appointed to be a Senior Fellow of the CSSI during the congress.

Professor Nagata reported on recent data from his group about ERdj5, an ER-resident disulfide reductase that has a central role in ER-associated degradation, known as ERAD. They found that ERdj5 is involved in calcium homeostasis via the regulation of a calcium ATPase, SERCA2b. Dr. Nagata also reported on a novel J-protein located in the ER, ERdj8, a negative regulator of macro-autophagy. Autophagy is also part of the retention of intracellular homeostasis through the constitutive degradation of abnormal proteins and organelles. ERdj8 is the first negative regulator of this process to be reported.

## CSSI medal session

The 2015 award of the medallion of the Cell Stress Society International recognizing career achievement was presented to Kazutoshi Mori (Kyoto University, Japan). The award was presented by CSSI President Tangchun Wu and Secretary-treasurer Lawrence Hightower. This was the seventh awarding of the medallion to an individual. The previous award winners are Takashi Yura (Japan) in 2000, Susan Lindquist (USA) in 2003, Aaron Ciechanover (Israel) in 2005, R. John Ellis (United Kingdom) in 2007, Costa P. Georgopoulos (USA) in 2009, and Ferruccio Ritossa (Italy) in 2010. The medallion is a bronze replica of the CSSI large seal and it was designed and forged in Hungary. The accompanying certificate recognized Professor Mori for “his discoveries of the functions and molecular mechanisms underlying the Unfolded Protein Response” (Ninagawa et al. 2015; Koga et al. 2015).

Kazutoshi Mori was born in Okayama. After graduating from the Okayama Prefectural Kurashiki-Seiryo high school, he entered the Faculty of Engineering of Kyoto University. In 1978, he moved to the Faculty of Pharmaceutical Sciences where he pursued master and doctoral studies on mannan-binding proteins and EGF in liver. Kazutashi received his PhD in 1987. In 1989, he moved to Dallas at the University of Texas Southwestern Medical Center for a post-doctoral with Drs. Mary Jane Gething and Joe Sambrook. It was during this period that Kazutoshi became interested in the ER stress and the induction of the KAR2 (Bip/grp78) gene in yeasts.

In 1993, he returned to Kyoto to join The HSP Research Institute founded by Prof Takashi Yura, incidentally the first winner of the CSSI Medallion. He became an associate professor in the Graduate School of Biostudies and is a professor in the department of Biophysiscs at the University of Kyoto.

For his medal address, Professor Mori gave a talk titled “Protein Quality Control by the Unfolded Protein Response” (Supplementary Figure 5). He gave a brief historical perspective of the discovery of the response beginning with the work by Mary-Jane Gething and Joe Sambrook in 1988 showing that accumulation of aberrant proteins in the endoplasmic reticulum (ER stress) activated transcription of ER chaperones. This in turn activated a response in the ER that increased its capacity to fold proteins. They named this homeostatic (now called proteostatic) response the Unfolded Protein Response (UPR). Dr. Mori joined their group in 1989 and began by analyzing the molecular mechanism of the UPR. Along with Peter Walter, Mori found that yeast UPR contains the IRE1-HAC1 pathway. Mori and David Ron then showed that XBP1 is a transcription factor located downstream from mammalian IRE1. They went on to identify PERK and ATF6 as the second and third mammalian ER stress sensors. The entire field of stress response biology is now moving toward a deeper understanding of the physiological significance of the response in both health and disease. Professor Mori is using the medaka, a small fish model for the vertebrate responses, to unravel the plethora of ER stress response proteins now known to exist, and to understand the physiology of the response.

Professor Mori has received numerous awards for his work on the Unfolded Protein Response. Among these is the Investigator award of the Japanese Biochemical Society (2006), the Osaka Science Award (2008), Canada’s Gairdner International Award (2009), the Medal of Honor with Purple Ribbon from the Emperor of Japan (2010), the Lasker Basic Medical Research Award, the Shaw prize in Life Science and Medicine (2014), and the CSSI Medallion (2015).

## Poster session

The poster session was well attended and the poster presentations were of high quality. It was difficult for the judges to select the student poster award winners. Here are the names of the twelve students chosen: Yi Zhang, Tianqi Tao, Hong Zhang, M.C. Vaaltyn, Felipe C. M. Zoppino, Zhusong Mei, Jie Dong, Ling Li, Jinling Zhang, Zhiqing Zhang, Hongyan Yang, and Daragh Cuskelly. (A photo of the awards presentation is shown in Supplementary Figure 6).

Yi Zhang’s poster and the others in this paragraph were part of the session titled Stress Biology: Physiological and Psychological Response. The poster was titled “NLRP3 Inflammosome Mediates Chronic Mild Stress-induced Depression in Mice Via Neuroinflammation” (Chun-Lei Jiang laboratory, Shanghai). Data were presented suggesting that the NLRP3 inflammosome, a multi-protein complex of the innate immune system that detects pathogen- and damage-associated molecular patterns, mediated depression induced by stress by a mechanism involving immune activation. Tianqi Tao’s poster was titled “The PERK/Nrf2 pathway is Involved in Thapsigargin and Tunicamycin-induced Endoplasmic Reticulum Stress (ERS)-related Apoptosis in H9C2 cells” (Xiuhua Liu laboratory, Beijing). The H9C2 cell line, derived from rat embryonic heart tissue, was used to study apoptosis using AnnexinV/PI double staining and TUNEL assays. TG treatment and TM treatment for 48 h induced apoptosis along with increased expression of calreticulin, PERK phosphorylation, and Nrf2 nuclear translocation along with upregulation of ATF4 and CHOP. The ERS inhibitors Tau and TUDCA had the opposite effect. Hong Zhang’s poster was titled “Glutathionylation of DnaK Provides a Link Between Oxidative Stress and the Heat Shock Response” (Sarah Perrett laboratory, Beijing). The data presented confirmed that the functions of DnaK were downregulated by this modification and may have facilitated the release of Sigma32 factor from DnaK. This change would activate the bacterial heat shock response, providing a link between oxidative stress caused by ATP depletion and thio modifications, e.g., glutathionylation, and the heat shock response. Zhiqing Zhang’s poster was titled “Histone Deacetylase Inhibitor Trichostatin A Improves Spatial Memory in Sleep Deprivation-Induced Cognitive Deficits in Rats” (Zhiqing Zhang laboratory, Tianjin). The authors showed that HDAC inhibition reversed sleep deprivation-induced reduction in histone acetylation, downregulation of BDNF expression along with impairment of hippocampal-dependent cognitive function measured using the Morris water maze task.

The other winners came from the very large session entitled Stress and Diseases. M.C. Vaaltyn’s poster was titled “The Interaction of STI1/HOP with RhoC” (A.L. Edkins laboratory, South Africa). Preliminary data were presented showing that STI1/HOP and RhoC interact directly in vitro to become part of a complex containing Hsp90 in HS578T breast cancer lysates. These observations increase our knowledge of the role of STI1 in the function of RhoC, which is an important regulator of cancer metastasis. This laboratory reported previously that STI1/HOP may have a role in cell migration by controlling cytoskeletal dynamics of cancer cells via mechanisms involving RhoC. Felipe Zoppino’s poster was titled “Regulation of HSPB1 (HSP27) by has-miR-214 in Breast Cancer” (D.R. Ciocca laboratory, Argentina). These investigators used MCF-7 (luminal A) cells as a breast cancer model. Their data indicated that the miRNA hsa-miR-214 increases levels of the tumor-enhancing proteins Akt and phosphorylated Akt. High levels of HSPB1 were associated with significantly increased survival in luminal A breast cancer patients. However, patients having increased levels of hsa-miR-214 had significantly poorer survival. Mei Zhusong’s poster was titled “The Apoptotic Regulatory Protein ARC (apoptosis repressor with caspase recruitment domain) Inhibiting Cardiomyocyte Hypertrophy Induced by Hypertension” (Lingjia Qian laboratory, Beijing). It is known that myocardial hypertrophy is related to mitochondrial function, but the details of this relationship have not been discovered. The authors presented data that suggests that CK2, ARC, and ROS constitute an anti-hypertrophic pathway in the heart. Jie Dong’s poster was titled “Hypoxic Enhanced High Expression of Vasorin in HepG2 Cell Might Be Involved in HUVEC Migration” (Shao Ningsheng Laboratory, Beijing). Evidence was provided that Vasorin, a putative product of cellular stress responses, may function as a pro-angiogenic mediator that is secreted by tumors as part of their adaptation to hypoxic environments. Cellular stress responses are known to have an important role in tumor development, a process promoted by hypoxia through responses including prosurvival reactions and elevated angiogenesis. Jinling Zhang’s poster was titled “Pro-inflammatory TNF-alpha and Oligodendrocyte Maturation Involved in Antidepressant-line Effect of Aspirin in Rats with Behavioral Depression Induced by Stress” (Qingjun Huang Laboratory, Shantou). Evidence continues to mount in support of the ideas that inflammation and neurodegeneration are associated with depression. The authors showed that TNF-alpha and oligodendrocyte maturation play roles in the antidepressant effects of aspirin. A rat model of depression induced by Chronic Unpredictable Mild Stress was used to investigate the effects of aspirin on depressive behavior. The antidepressant and neurobiological effects of aspirin shown here suggest that aspirin and aspirin-like drugs should be pursued as potential therapeutics. Hong-yan Yang’s poster was titled “Berberine Ameliorates Oxidative Stress And Mitochondial Dysfunction Via Reducing Protein O-GlcNAcying In High Glucose-Induced HUVECs” (Ling Dong Laboratory, Xi’an). Protein covalent alterations by O-GlcNAc glycosylation are involved in oxidative stress within cells which in turn inhibits insulin receptor signaling in diabetic patients. Berberine is an isoquinoline alkaloid used in Chinese medicine. It is known to decrease cardiovascular impairment in diabetes in a pathway including activation of AMP-activated protein kinase. The authors provided evidence that berberine in an AMPK-dependent fashion reduced oxidative stress and mitochondrial dysfunction by decreasing O-GlcNAc concentrations in human umbilical vein endothelial cells induced with high levels of glucose. Daragh Cuskelly’s poster was titled “Counteracting Oxidative Stress: Developing Budding Yeast as a Cell Factory for Production of the Antioxidant Ergothioneine” (Gary W. Jones Laboratory, Ireland). Ergothioneine (EGT) is a sulfur-containing amino acid modified naturally. Why develop a process to make it efficiently and economically? It has applications as a food additive to prevent auto-oxidation and is also useful in the treatment/prevention of diseases associated with oxidative stress. The authors showed for the first time that *Saccharomyces cerevisiae* can produce EGT. They are currently working to scale up the process.

The last poster awardee comes from Session 13: Stress and Women’s Health. Ling Li’s poster was titled “Identification And Characterization Of Stress Chaperone Mortalin As A New Regulator Of Melanogenesis” (Renu Wadhwa Laboratory, Japan). Four rounds of screening in human melanoma G361 cells were used to identify 40 gene targets involved in human melanogenesis. Bioinformatic and pathway analysis showed that these gene targets are involved in regulation of cell proliferation, apoptosis, stress responses, and mitochondrial functions. The role of the mitochondrial stress chaperone mortalin in melanogenesis was discovered in this way.

## Cultural activities

The CSSI International Congresses have always included cultural activities to allow the participants to experience the culture of the host country. The meeting site, Huangshan City, is one of the most beautiful ancient cities in China, and it is home to two UNESCO World Heritage Sites. Mount Huangshan is known as the most beautiful mountain in China with its grotesque rock formations, its uniquely shaped pine trees growing out of cliffs, and its marvelous seas of clouds. The beautiful environment and traditional Chinese culture, including the Chinese opera and Tai Chi, demonstrated by a professor from Tianjin University of Sport, reduced our stress, helped our health, and created the perfect environment for academic communication and cooperation. Many scientific collaborations and student exchanges have happened because of these cultural events shared together by the congress participants.

## Concluding remarks

The congress was attended by a good mixture of students, post-doctoral fellows along with young and senior investigators. In this regard, it fulfilled the current theme of the CSSI Executive Council to nurture the next generation of stress response researchers. The specific goal of this congress was to foster stronger professional relationships among Chinese and international scientists in our field. This has been facilitated by the rapid development of stress medicine in China in recent years. This was evidenced during the congress by the English fluency and high quality of work presented by Chinese scientists and their students in oral presentations and posters. The strong working relationship that was formed between our two organizations, CSSI and the committee of Stress Physiology, CAPS, during the organization of this congress, no doubt will continue long into the future, strengthening our field.

Work on the Eighth CSSI Congress is already underway. It is scheduled for Turku, Finland, in August of 2017. Professor Lia Sistonen is the principal organizer. Up-to-date information about this congress is at the CSSI website www.cellstressresponses.org. Please join us!

## Electronic supplementary material

Below is the link to the electronic supplementary material.

Supplementary Figure 1 Official photograph of Congress participants, over 200 scientists and their students from 22 countries. (JPG 1730 kb)

Supplementary Figure 2 Keynote Speaker Fu-Chu He. (JPG 1084 kb)

Supplementary Figure 3 Keynote Speaker E.R. (Ron) de Kloet. (JPG 1010 kb)

Supplementary Figure 4 Keynote Speaker Kazuhiro Nagata. (JPG 590 kb)

Supplementary Figure 5 CSSI Medallion recipient for 2016, Kazutoshi Mori. (JPG 1965 kb)

Supplementary Figure 6 Recipients of the Student Poster Award at the presentation ceremony along with Principal Organizer Lingjia Qian and Session Chairman Larry Hightower. (JPG 1691 kb)

Supplementary Figure 7 Congratulations and a gift to commemorate an excellent congress presented to Prinicipal Organizer Lingjia Qian by her co-organizers Robert M. Tanguay, Larry Hightower and CSSI President Tangchun Wu. (JPG 2378 kb)
