# Supplementary figures and images for: Stress and health Huangshan-style
Source: Cell Stress Chaperones. 2016 Feb 4;21(3):373–8. doi: 10.1007/s12192-016-0674-8 (PMC4837187; doi:10.1007/s12192-016-0674-8)

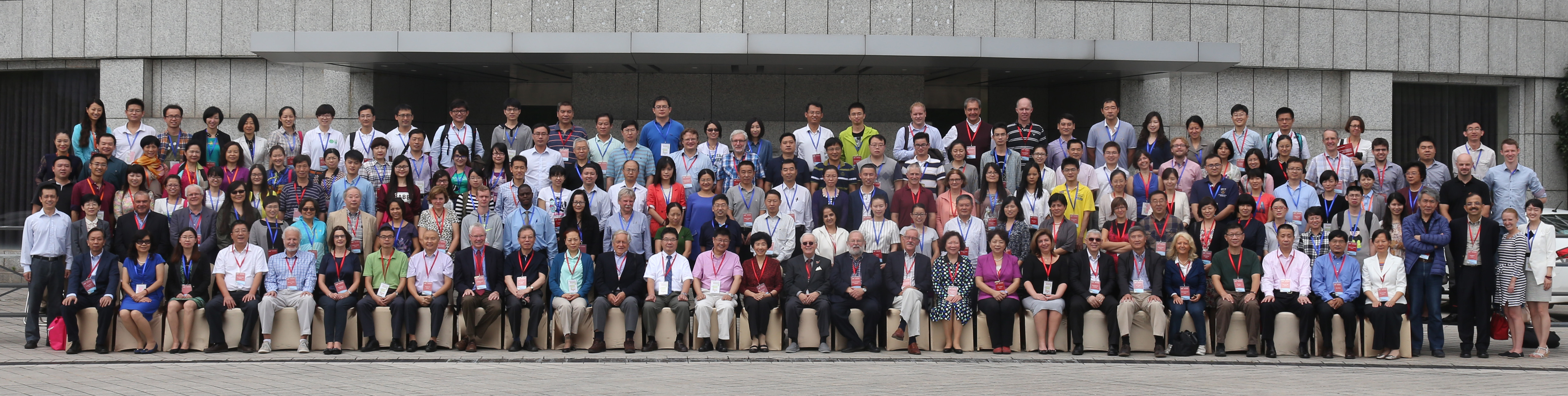

Supplement: Supplementary file 1 — Official photograph of Congress participants, over 200 scientists and their students from 22 countries. (JPG 1730 kb) [file 12192_2016_674_Fig1_ESM.jpg]

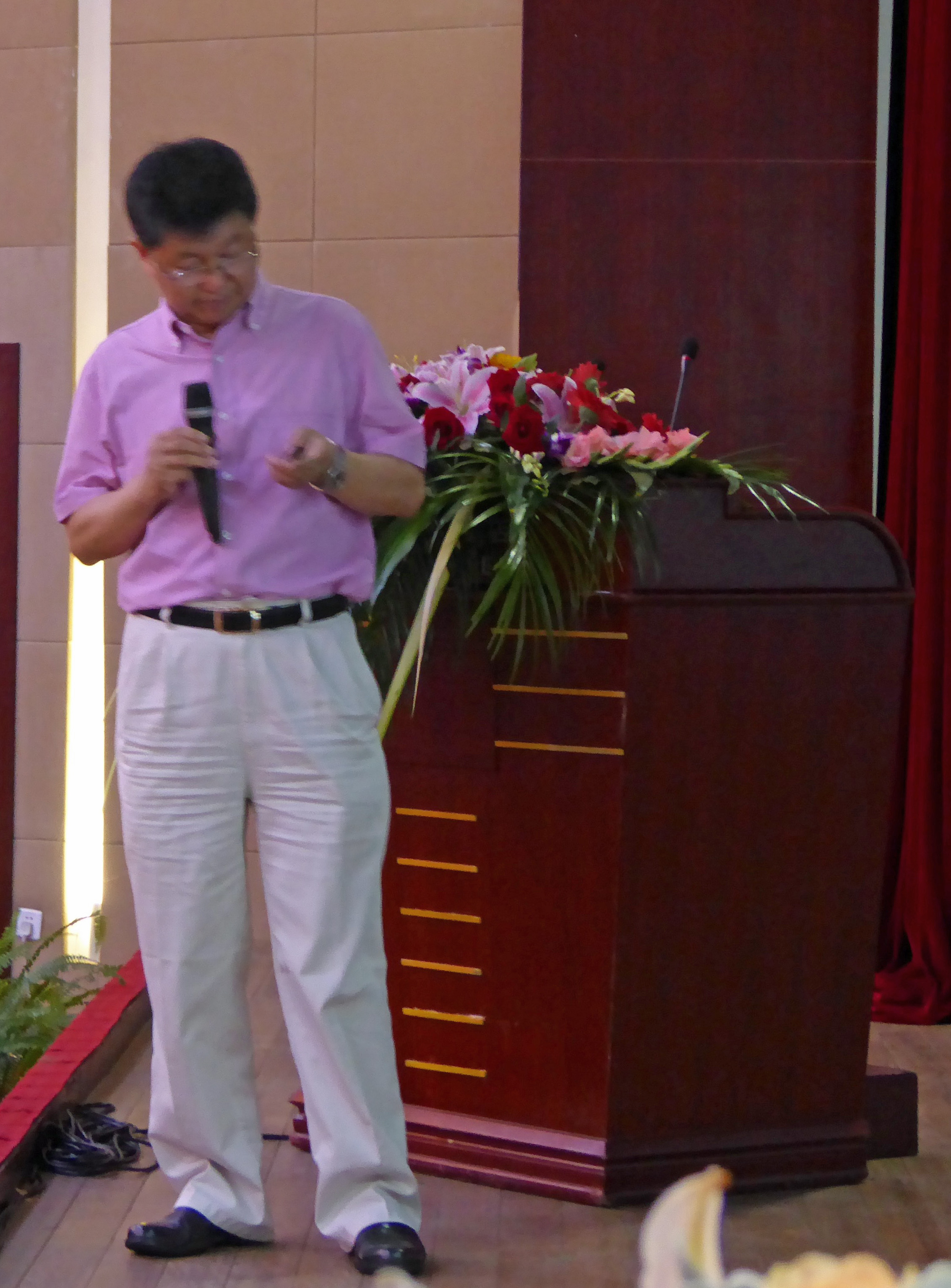

Supplement: Supplementary file 2 — Keynote Speaker Fu-Chu He. (JPG 1084 kb) [file 12192_2016_674_Fig2_ESM.jpg]

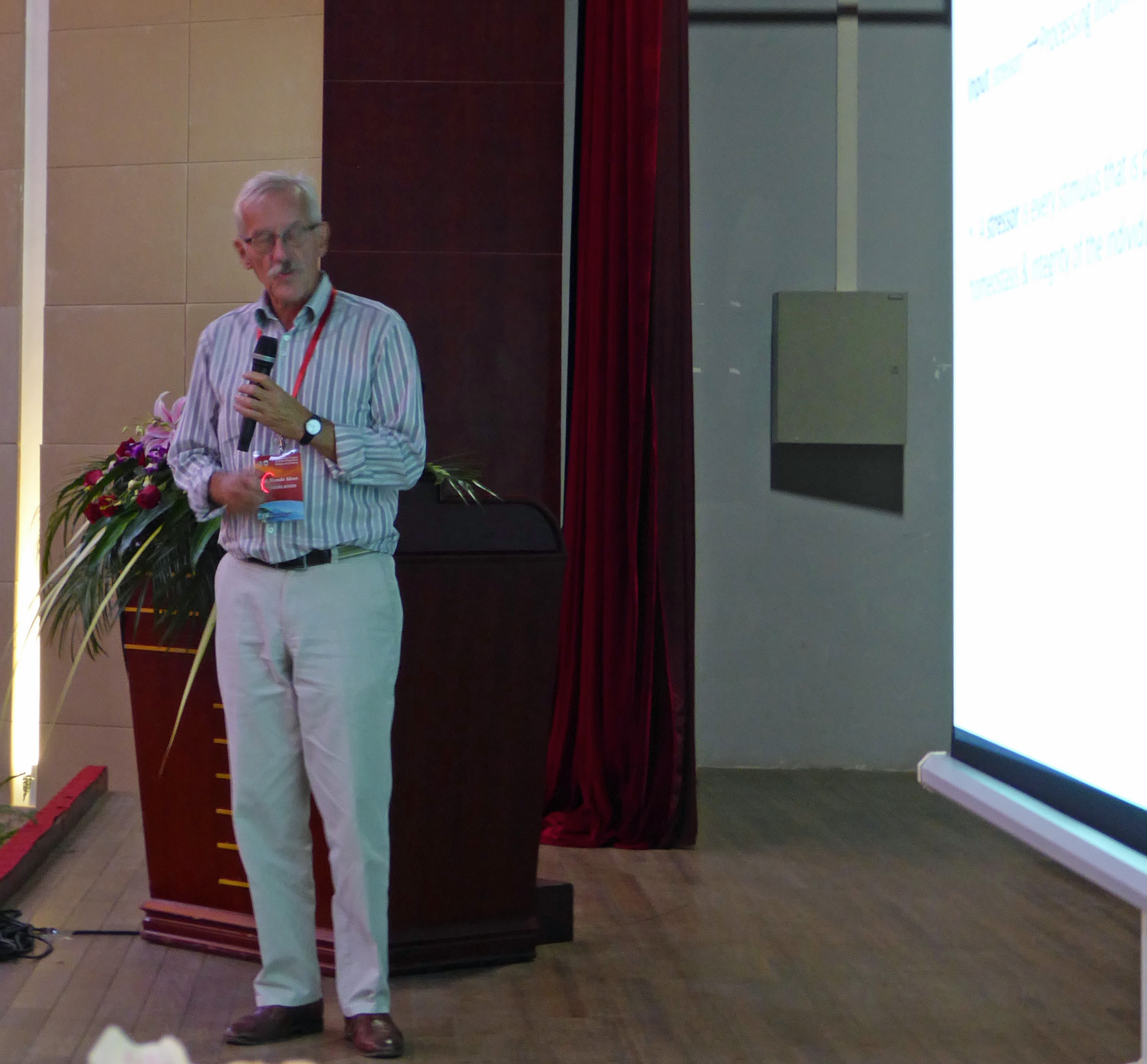

Supplement: Supplementary file 3 — Keynote Speaker E.R. (Ron) de Kloet. (JPG 1010 kb) [file 12192_2016_674_Fig3_ESM.jpg]

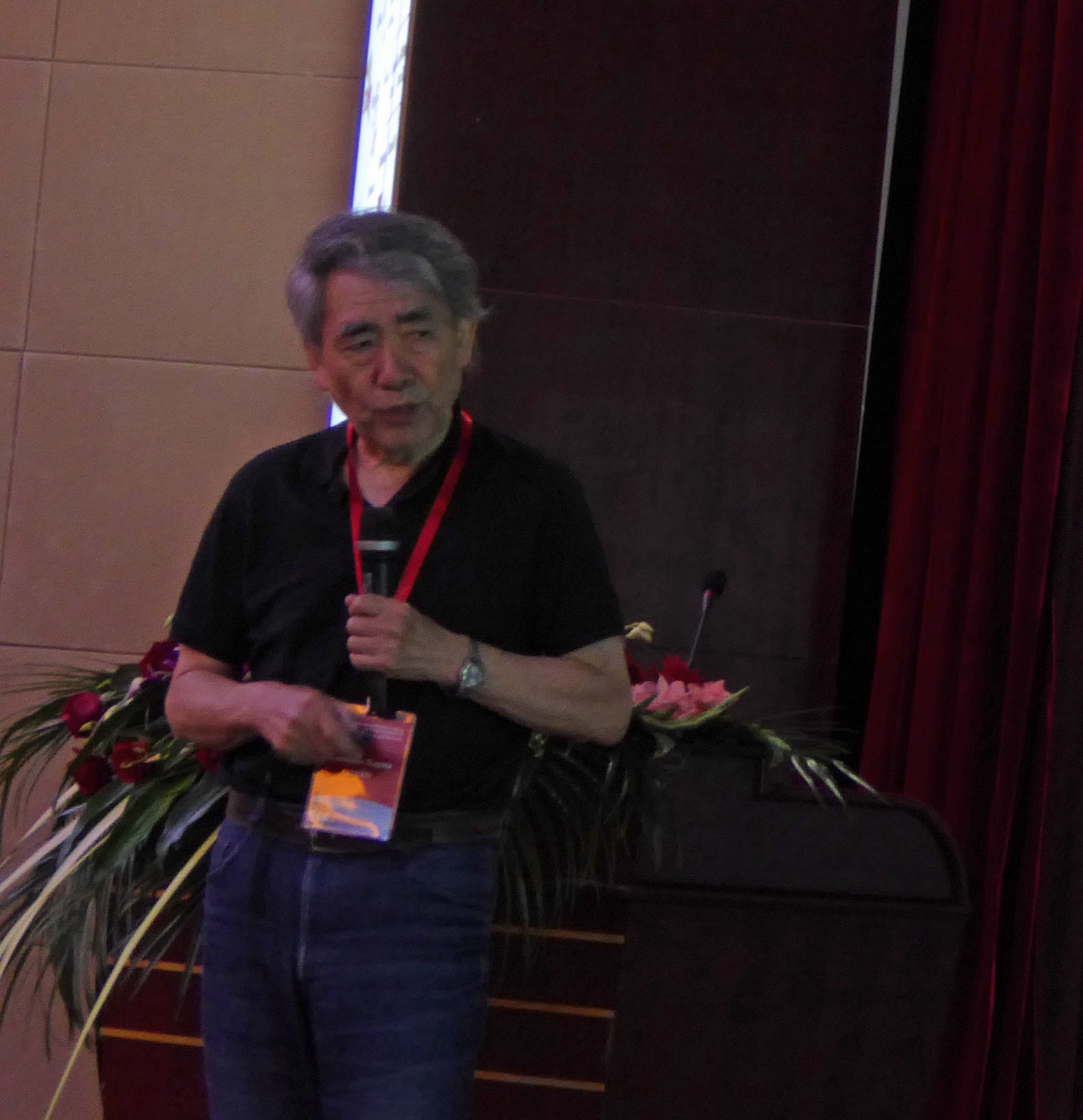

Supplement: Supplementary file 4 — Keynote Speaker Kazuhiro Nagata. (JPG 590 kb) [file 12192_2016_674_Fig4_ESM.jpg]

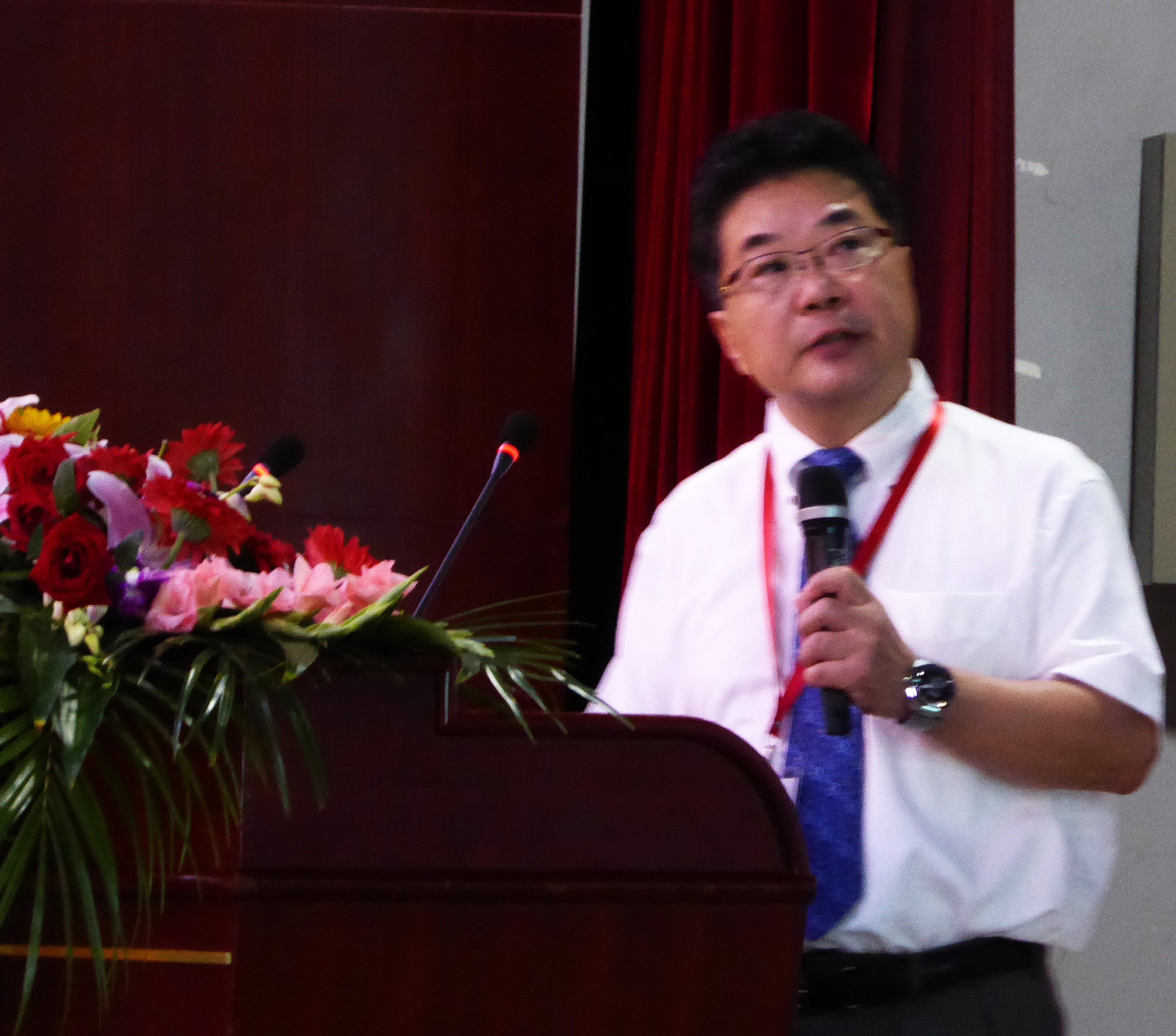

Supplement: Supplementary file 5 — CSSI Medallion recipient for 2016, Kazutoshi Mori. (JPG 1965 kb) [file 12192_2016_674_Fig5_ESM.jpg]

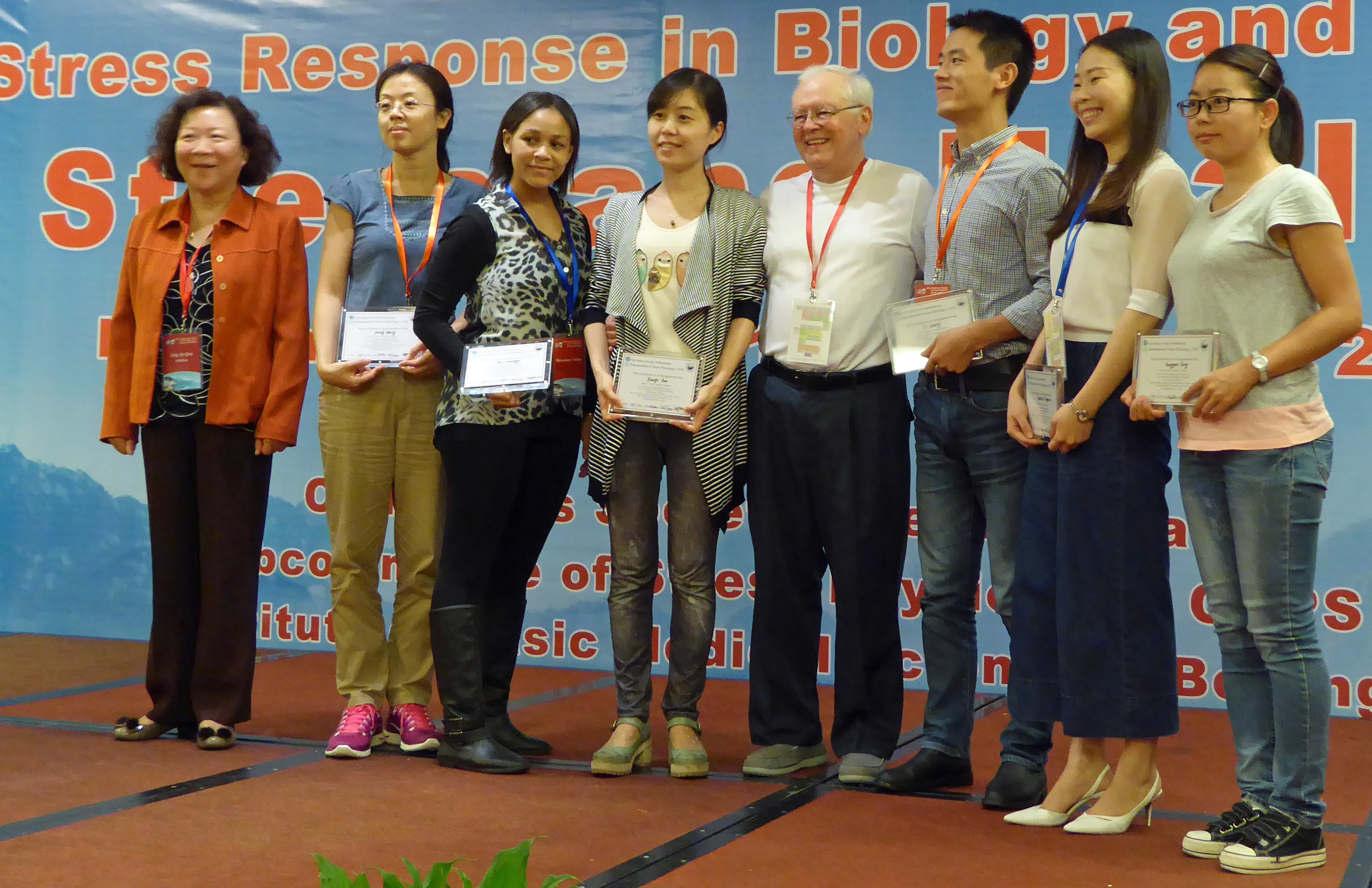

Supplement: Supplementary file 6 — Recipients of the Student Poster Award at the presentation ceremony along with Principal Organizer Lingjia Qian and Session Chairman Larry Hightower. (JPG 1691 kb) [file 12192_2016_674_Fig6_ESM.jpg]

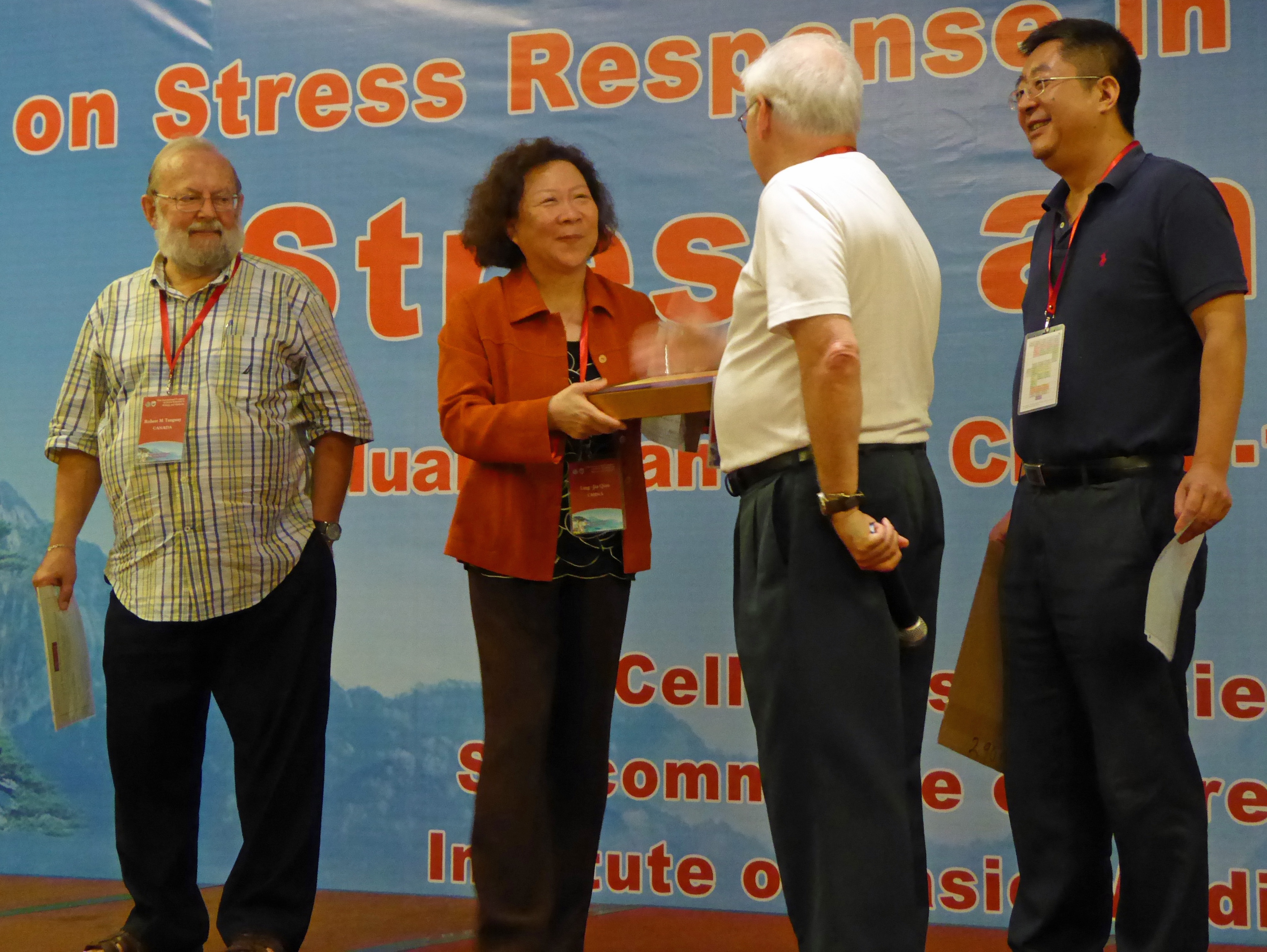

Supplement: Supplementary file 7 — Congratulations and a gift to commemorate an excellent congress presented to Prinicipal Organizer Lingjia Qian by her co-organizers Robert M. Tanguay, Larry Hightower and CSSI President Tangchun Wu. (JPG 2378 kb) [file 12192_2016_674_Fig7_ESM.jpg]
